# *Lactobacillus helveticus*: importance in food and health

**DOI:** 10.3389/fmicb.2014.00338

**Published:** 2014-07-04

**Authors:** Giorgio Giraffa

**Affiliations:** Consiglio per la Ricerca e la Sperimentazione in Agricoltura, Centro di Ricerca per le Produzioni Foraggere e Lattiero-CasearieLodi, Italy

**Keywords:** *lactobacillus helveticus*, dairy products, health benefits, dairy microbiology, probiotics

*Lactobacillus helveticus* belongs to a group of organisms collectively known as lactic acid bacteria (LAB). This organism has a Generally Recognized as Safe (GRAS) status and displays a number of features that make it particularly suitable for dairy applications. *Lactobacillus helveticus* is traditionally used in the manufacture of Swiss-type cheeses and long-ripened Italian cheeses such as Emmental, Gruyere, Grana Padano and Parmigiano Reggiano and it is the prevalent species recovered from natural lactic starter cultures used for the production of typical Italian cheese. *Lactobacillus helveticus* is gaining also importance as health-promoting culture in probiotic and nutraceutic food products. It has the potential to produce bioactive peptides or bacteriocins, and exert synbiotic effect when associated with prebiotics in fermented dairy products. *Lactobacillus helveticus* can therefore be considered as a multifunctional LAB with increasing importance in the food industry. This fact, together with the relevant literature on this topic, were the elements behind the choice to implement a research topic on *L. helveticus*.

The present topic includes four review articles. Cremonesi et al. (2013) reported on the recent genome sequencing to completion of five *L. helveticus* strains and the sequence comparison with other genomically characterized lactobacilli. Interestingly, the genomic analysis of the first sequenced strain, *L. helveticus* DPC 4571, isolated from cheese and selected for its characteristics of rapid lysis and high proteolytic activity, revealed a plethora of genes with industrial potential, including those responsible for key metabolic functions such as proteolysis, lipolysis, and cell lysis (Slattery et al., 2010). These genes and their derived enzymes underline the well known propensity of *L. helveticus* to be used as dairy starter in the production of cheese and cheese derivatives. To this regard, the review of Griffiths and Tellez (2013) described more specifically the role of the proteolytic system in *L. helveticus*. *L. helveticus* is among the most nutritionally fastidious LAB, requiring 14 exogenous aminoacids (Chopin, 1993). To assure its nutritional requirements when grown in milk, *L. helveticus* relies on a potent proteolytic system capable of producing short peptides and liberating amino acids from casein (Callanan et al., 2008). This explains why it has higher proteolytic activity than most other lactobacilli. The proteolytic system of *L. helveticus* consists mainly of cell envelope proteinases which initially cleave caseins to large peptides, intracellular peptidases further degrading peptides to small peptides and amino acids, and specific transport proteins which transfer amino acids and peptides across the cytoplasmic membrane (Slattery et al., 2010).

Cremonesi et al. (2013) mentioned the potential of *L. helveticus* to produce peptides with a biological function, such as those possessing an inhibitory activity on the angiotensin converting enzyme (ACE), demonstrating the therapeutic value of this species when used in fermented dairy products. These peptides have been shown in clinical trials (Jauhiainen et al., 2005). Intriguingly, comparative genomics showed the remarkable similarity in gene content of *L. helveticus* with many intestinal lactobacilli, especially for key gene sets facilitating an adaptation to food matrices or the gastrointestinal tract (Slattery et al., 2010). The same conclusions can be drawn by the review of Griffiths and Tellez (2013) who reported that various peptides with physiological functions, such as immunostimulating peptides, antimicrobial peptides, opioid peptides, mineral binding peptides and antihypertensive peptides, can be isolated from products fermented with *L. helveticus*. The health-promoting properties of *L. helveticus* were reviewed by Taverniti and Guglielmetti (2013) who summarized the wide literature concerning the ability of this species to positively influence human health. Also according to the results from the comparative genomics, it is not surprising that *L. helveticus* possesses many commonly recognized probiotic features, such as the ability to survive gastrointestinal transit, adhere to epithelial cells, and antagonize pathogens. *L. helveticus* is also able to prevent gastrointestinal infections, enhance protection against pathogens, modulate host immune responses, and affect the composition of the intestinal microbiota (Slattery et al., 2010). No less important is the indirect benefit brought by this bacterium to the human host in term of enhancement the bioavailability of nutrients and removal allergens and other undesired molecules from food (Taverniti and Guglielmetti, 2013).

The topic is concluded by a minireview summarizing the technological and probiotic potential of BGRA43, a human intestinal isolate with antimicrobial activity especially against *Yersinia enterocolitica*, *Shigella sonnei*, *Shigella flexneri*, and *Streptococcus pneumoniae* (Strahinic et al., 2013). Interestingly, BGRA43 embodies many of the useful properties of *L. helveticus* such as proteolytic activity on both casein and β-lactoglobulin, ability to release bioactive peptides in fermented milk, modulate the production of proinflammatory cytokines IL-6 and TNF-α, and BGRA43, and survive in simulated gastric and intestinal conditions. In conclusion, *L. helveticus* is a polyfunctional lactic acid bacterium with important implications in dairy biotechnology. To meet the industry demand for product diversification, new interesting strains should be searched and characterized to design cultures with expanded properties to be applied in fermented dairy products. To this regard, *L. helveticus* opens exciting perspectives for industry-driven applications in cheese ripening or health-promoting cultures.

## Conflict of interest statement

The author declares that the research was conducted in the absence of any commercial or financial relationships that could be construed as a potential conflict of interest.
